# Central Vein Thrombosis in Pulmonary Neuroendocrine Neoplasm: A Novel Presentation

**DOI:** 10.7759/cureus.16499

**Published:** 2021-07-19

**Authors:** Adnan Liaqat, Awais Farooq, Qamar Gulzar, Stephanie Stalcup, Bsmah Abdalsalam

**Affiliations:** 1 Internal Medicine, Southeast Health Medical Center, Dothan, USA

**Keywords:** central vein thrombosis, neuroendocrine tumor, lung malignancy, internal jugular vein thrombosis, subclavian vein thrombosis

## Abstract

Internal jugular vein (IJV) thrombosis is a rare finding and is usually associated with central venous catheterization, neck infections, or local trauma. Neuroendocrine tumors (NETs) rarely predispose to central vein thrombosis. The usual presentation of pulmonary NET depends on tumor location and is usually non-specific. It ranges from asymptomatic to cough, hemoptysis, dyspnea, etc. Here we present the case of a 52-year-old male with right-sided neck swelling. Ultrasound imaging of the neck revealed right IJV and right subclavian vein thrombosis. Further imaging with computed tomography (CT) scan of the chest showed mediastinal mass. Histopathology findings were consistent with NET of pulmonary origin. Patient was started immediately on anti-coagulation and radiology oncology was consulted for tumor-specific treatment. This case highlights an association of central vein thrombosis with underlying mediastinal and lung malignancies.

## Introduction

Lung cancer is one of the leading causes of death among males worldwide. Malignancy significantly increases the risk of venous thromboembolism in patients because of a hypercoagulable state. Isolated intravascular thrombosis, however, is uncommon and cancer-related superior vena cava (SVC) thrombosis is found in only 0.04% of hospitalized adults [1]. The incidence and prevalence of neuroendocrine tumor (NET) has increased over the period of many years in the recent past [2]. The gastrointestinal tract (62%-67%) and the lung (22%-27%) are the most frequent primary sites of NET [3]. NETs rarely predispose to central vein thrombosis. It is a markedly fatal complication, and timely anti-coagulation is crucial. Compared to other areas commonly involved in deep venous thrombosis associated with neoplastic process, internal jugular vein (IJV) is relatively rare [4]. This case highlights the unique finding of IJV and subclavian vein thrombosis in a patient with underlying NET of pulmonary origin. 

## Case presentation

The patient is a 52-year-old male who presented with right-sided neck pain with associated localized swelling. He did not have any significant past medical history. Symptoms began approximately one month ago and had gradually worsened. On further questioning, the patient also complained of a mild intermittent non-productive cough for about a month. 

**Figure 1:**
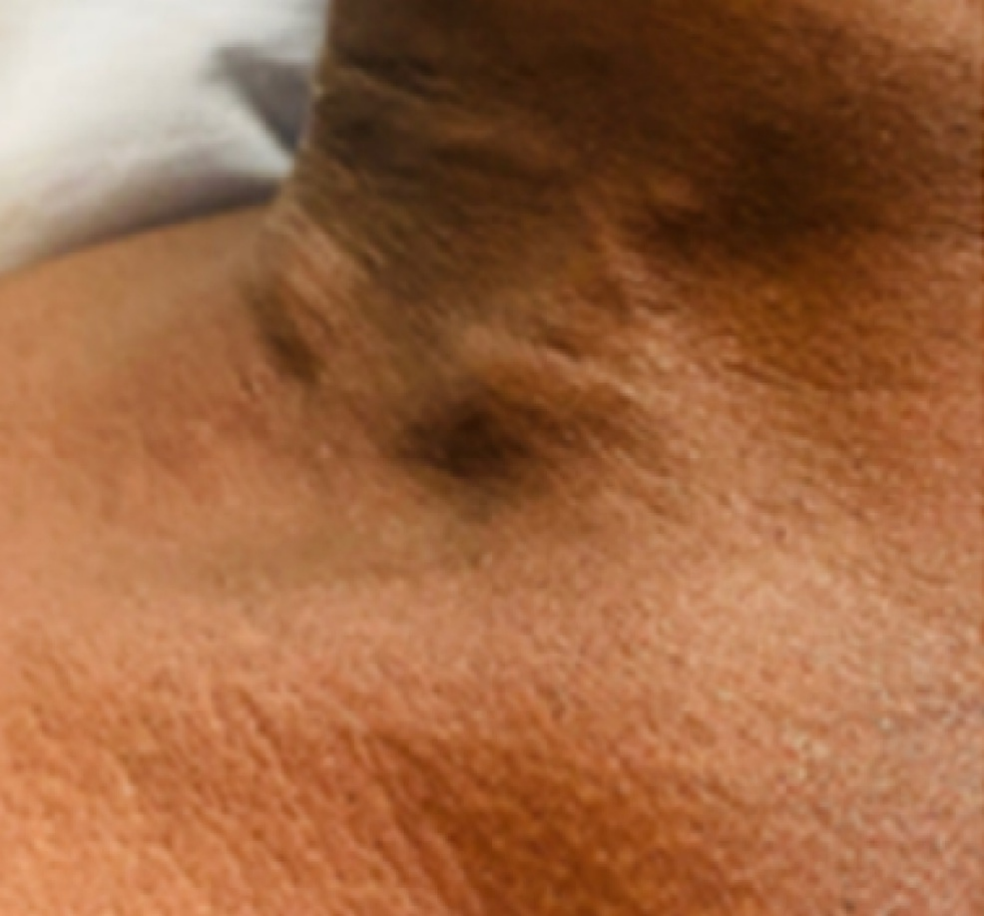
Image showing right-sided neck swelling

Physical examination revealed two small areas of swelling on the right side of the neck when the patient’s head turned to the left (Figure 1). There was no evidence of facial edema, significant dilation of veins in the neck, difficulty swallowing, visual disturbances, or difficulty breathing at the time of initial presentation.

**Figure 2:**
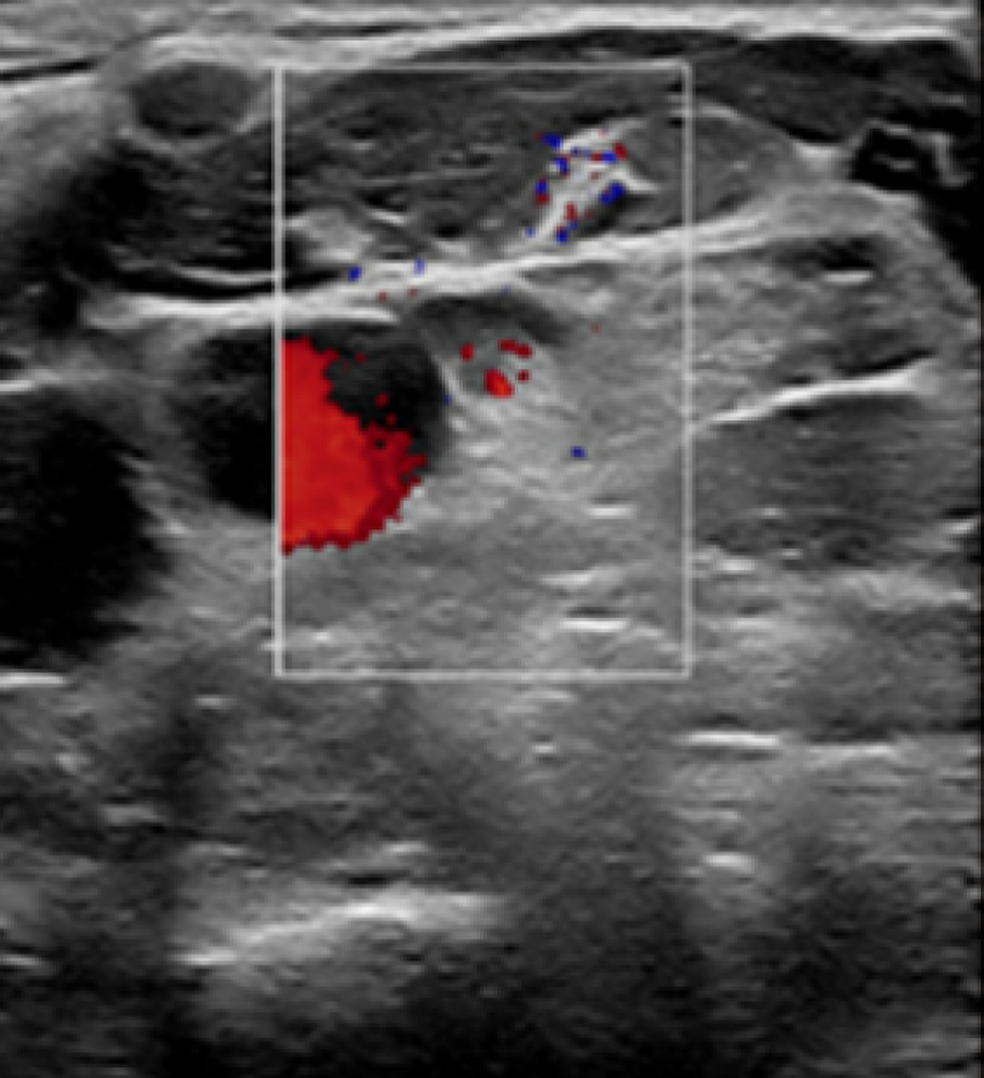
Doppler ultrasound image of the right side of neck Highlighted portion in the image shows non-occlusive thrombus in the right jugular and subclavian veins.

Labs revealed mild leukocytosis with predominant neutrophils. Ultrasound imaging of the neck showed an extensive non-occlusive thrombus in both the right jugular and right subclavian veins (Figure 2). Patient was started on anti-coagulation with intravenous heparin immediately.

**Figure 3:**
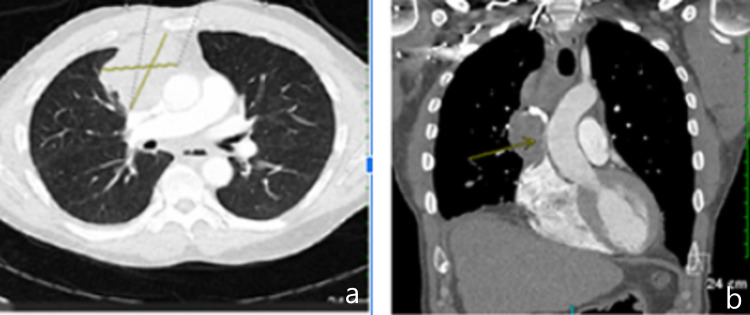
Computed tomography (CT) scan of the chest (a) Axial section with cross mark showing mediastinal mass. (b) Coronal section with arrow pointing to mediastinal mass.

A computed tomography (CT) scan of the chest showed widespread malignancy with multifocal, pathologic mediastinal lymphadenopathy resulting in moderate- to high-grade SVC obstruction without classical clinical presentation (Figure 3).

**Figure 4:**
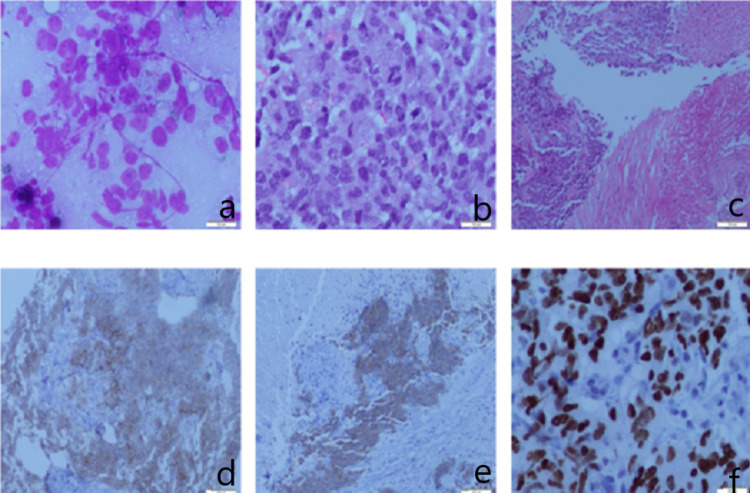
Histopathology and immunohistochemistry staining (a) Malignant cells, high nucleus-to-cytoplasm ratio. (b) Mitotic figures, neoplastic proliferation. (c) Broad zone of necrosis.  (d) CD56-positive staining. (e) Synaptophysin and chromogranin stain positive. (f) Nuclear stain TTF-1 positive.

Mediastinal CT-guided fine-needle biopsy was performed. Histologic sections of biopsy showed results consistent with a poorly differentiated neuroendocrine carcinoma. On immunohistochemical analysis, cells were CD-56 positive, S-100 positive, synaptophysin positive, and TTF-1 positive (Figure 4), which were all clear indications of an NET of pulmonary origin. MRI brain and CT abdomen were both negative for metastatic lesions. Radiation oncology was consulted, and the patient subsequently underwent two radiotherapy sessions prior to discharge with plans to start outpatient chemotherapy. The patient was then transitioned to oral anti-coagulation with rivaroxaban.

## Discussion

Data describing the association between central vein thrombosis with underlying pulmonary malignancies is limited in the current literature. Our case focuses on a rare association of central venous thrombosis with NETs. Identification of NET based on the presenting complaints can be a challenge, as NET presentation can range from asymptomatic to non-specific symptoms. Several of the common symptoms can also overlap with other respiratory conditions (such as asthma, chronic obstructive pulmonary disease) making it difficult to differentiate [5]. Tumor location is usually a determining factor for symptoms. Centrally located tumors usually present with compressive symptoms related to tumor burden including cough, hemoptysis, wheezing, and obstructive pneumonia, whereas peripherally located tumors are usually incidental findings [5]. Malignancy is an established risk factor for venous thromboembolism. Although the pathophysiology is not completely understood, production of certain procoagulant/fibrinolytic substances and the release of inflammatory cytokines along with physical interaction of tumor cells with various blood cells are thought to be the primary mechanism involved in development of venous thromboembolism [6]. Hemodynamic compromise (i.e., stasis), especially in the setting of compression due to mass effect of tumor, can also be a contributing factor [6]. In our case, the adjacent tumor caused compression of SVC. Because of the relatively brisk blood flow through the SVC, it is an unusual site for thrombosis [1]. IJV and subclavian vein are relatively rare locations to develop thrombosis but stasis likely contributed to development of central vein thrombosis in our patient because of distal SVC compression. The interesting fact about our case is that central vein thrombosis led to the diagnosis of NET of pulmonary origin. In a patient with central vein thrombosis, focus should be on further investigations to rule out any underlying mediastinal or pulmonary malignancies. Once the underlying neoplastic process is identified, consideration of therapeutic anti-coagulation and focus on tumor-specific treatment should be the immediate next step in management of such complicated cases. 

## Conclusions

Malignancy is a known risk factor for venous thromboembolism. Once we find central vein thrombosis, we should focus further investigation on possible underlying neoplastic process, particularly involving lung or mediastinum. Diagnostic imaging with CT scan, interventional radiology-guided biopsy, or endobronchial biopsy can all be used to identify the primary lesion. Therapy with anti-coagulation can be considered and tumor-specific treatment can be started once tissue diagnosis is established. 
